# A 16-week school-based intervention improves physical fitness in Slovenian children: a randomized controlled trial

**DOI:** 10.3389/fphys.2024.1311046

**Published:** 2024-01-18

**Authors:** Tanja Petrušič, Dario Novak

**Affiliations:** ^1^ Faculty of Education, University of Ljubljana, Ljubljana, Slovenia; ^2^ Faculty of Kinesiology, University of Zagreb, Zagreb, Croatia

**Keywords:** children, sport, physical fitness, games, athletics, gymnastics, school intervention

## Abstract

**Introduction:** The aim of this study was to evaluate the effects of a 16-week school-based physical activity (PA) intervention on physical fitness (PF) (speed, hand-eye coordination, flexibility) of 8– to 9-year-olds.

**Methods:** A total of seventy-eight boys and girls (boys: *n* = 45, aged 8.4 ± 4.9 years; girls: *n* = 42, aged 8.6 ± 0.5 years) from a school in Slovenia were randomly assigned to either a group with an after-school PA program (EXP) or a control group (CON) that participated exclusively in mandatory physical education (PE). The EXP group engaged in the extracurricular PA program for 60 min twice a week for 16 weeks, concurrent with regular PE classes. The program primarily involved elementary PE games that included elements of athletics (e.g., skipping, push- off running, hopping, crossstepping, and jumping) and gymnastics (e.g., handstand, forward roll, backward roll, hand support jumps, squat jump on a vault box, climbing on horizontal bars, incline benches and ropes, crawling, and jumping rope). Standardized tests appropriate for this age group were used to assess PF, including the sit and reach test (SAR), the 30-meter sprint, and the alternate hand wall toss test at distances of 1.0 and 2.0 m (AHWT 1.0 and 2.0).

**Results:** There was a significant group-time interaction for SAR test (EXP group increase: +1.6 cm, +6.3%; CON group decrease: −0.1 cm, −0.4%; *p* < 0.001, ηp^2^ = 0.361), and the 30 m sprint (EXP group improvement: −0.4 s, −6.3%; CON group decrease: +0.1 s, +1.6%; *p* < 0.001, ηp^2^ = 0.193). Similarly, the EXP group improved by +2.1 points (+25.6%) in the 1.0 m wall throw with the alternating hand, while the CON group showed only minimal changes (−0.2 points, −2.4%; *p* < 0.001, ηp^2^ = 0.545). No significant interaction was found for the 2.0 m toss (EXP and CON group both −0.1 points, −2.6%; *p* = 0.888, ηp^2^ = 0.001). *Post-hoc *analyses with paired t-tests revealed that the EXP group showed significant improvements in SAR test (*p* < 0.001), 30 m sprint (*p* < 0.001) and AHWT 1.0 test (*p* < 0.001), while the CON group showed no significant changes in SAR test (*p* = 0.533), 30 m sprint (*p* = 0.150), AHWT 1.0 test (*p* = 0.186) and AHWT 2.0 test (*p* = 0.430).

**Discussion:** The results of the study showed that the extracurricular program with only two additional weekly sessions significantly improved the components of PF in 8- to 9-year olds. Significant improvements were observed in the areas of flexibility, speed and coordination, as shown in the SAR test, 30-meter sprint and 1.0-meter handwall toss tests. However, no similar improvements were observed in the 2.0-meter handwall toss, which illustrates the specific areas of impact of the program.

## 1 Introduction

Improving the physical fitness (PF) of school-age children is an important endeavour that lays the foundation for a healthier lifestyle and has lasting effects on their overall wellbeing (Bermejo-Cantarero et al., 2023). Among all the components of PF, speed, hand-eye coordination and flexibility are critical attributes that contribute to children’s physical performance, cognitive development and activities of daily living (Liu et al., 2022). While these components appear to have a relatively small impact on health, future outcomes and disease prevention, they play an important role in the development of 8– to 9-year-olds, contributing to their physical performance, cognitive development and activities of daily living.

During the transition from early to middle childhood, 8–to 9-year-olds undergo significant physical growth and maturation (Aliriad et al., 2023). This developmental period is characterized by the acquisition of basic PF skills that serve as a foundation for more complex movements and activities (OBrien et al., 2016; Teich et al., 2023). Compared to some recent studies by various authors (Bauer et al., 2022; Kryeziu et al., 2023; Mohammadi-Nia et al., 2023; Osipov et al., 2023; Wu et al., 2023; Yuksel et al., 2020) that have examined different exercise programs in children and their different effects on PF, the importance of understanding these relationships in the context of child development is becoming increasingly clear. Speed is a central component of PF and refers to the ability to perform movements quickly and efficiently (Barbieri et al., 2022; Fernandez-Fernandez et al., 2023; Jafar et al., 2023; Volk et al., 2023). Although it may appear to make a smaller contribution to health and long-term disease prevention, the importance of speed should not be underestimated, especially in the developmental phase of 8–to 9-year-olds (Herszage et al., 2023). Promoting speed in children at this crucial developmental stage goes beyond pure physical performance. It also plays a fundamental role in cognitive processes, including decision-making and motor responsiveness (Lin et al., 2023). Refining 8–to 9-year-olds’ speed-related motor skills leads to improved reaction times and more efficient movement execution. This in turn enables them to participate in activities that require quick reactions, such as team sports and games (Herszage et al., 2023). It is important to emphasise that the development of speed in 8–to 9-year-olds not only contributes to their PF, but also has a lasting effect on their cognitive development. When children engage in activities that challenge their speed-related PF, it not only boosts their physical confidence but also their overall enthusiasm for physical engagement (Yamada et al., 2021; Schirmer et al., 2023). Hand-eye coordination is another essential element of PF that requires the seamless integration of muscle groups to perform precise and controlled movements (Lima et al., 2023; Tang et al., 2023). As 8–to 9-year-olds refine their hand-eye coordination, they gain the ability to perform complex movements with greater accuracy and fluidity. This improvement is due to the continued refinement of neural pathways that facilitate communication between the brain and muscles (Demers and Levin, 2017; Urai et al., 2022; Park and Jeong, 2023). As a result, 8–to 9-year-olds can engage in activities that require intricate hand-eye and motor coordination, such as dance, team sports, and various forms of artistic expression (Ludyga et al., 2022; Pastorek Gripson et al., 2022; Tao et al., 2022). Furthermore, it is crucial to recognise that hand-eye coordination plays a critical role in the development of fine and gross motor skills that are essential for everyday tasks and school activities. Children’s ability to perform these tasks with greater accuracy and coordination can have a lasting impact on their academic progress and general wellbeing (Giuriato et al., 2022; Navarra et al., 2022; Cinar et al., 2023; Kojić et al., 2023). Flexibility, even if it appears to make a lesser contribution to health, future outcomes and disease prevention, is an important facet of PF that deserves attention. Flexibility refers to the range of motion of the joints and the suppleness of the muscles and tendons (Konrad et al., 2022). During the eighth to ninth year of life, flexibility increases significantly due to progressive growth and adaptation of the musculoskeletal system (Zhang et al., 2023). This increased flexibility allows 8–to 9-year-olds to perform a wider range of movements while reducing the risk of overuse or injury (Yang et al., 2022; Behm et al., 2023; Zvetkova et al., 2023). Activities that promote flexibility, such as stretching and yoga, can help maintain healthy joints and muscles while promoting awareness of the importance of regular physical activity (PA) (Chan et al., 2023; Y; Cho et al., 2023). Developing flexibility during this stage lays the foundation for more agile and adaptable physicality, which is important for both recreational and functional movements (Tsao et al., 2022; Zinelabidine et al., 2022). Targeted interventions at this stage therefore have the potential to positively impact children’s PF and promote a lifelong appreciation for PA.

This study examined the effects of a 16-week school-based PA intervention on the PF of 8–to 9-year-olds, focusing on three selected components of PF: speed, hand-eye coordination, and flexibility. Although these components have a relatively small impact on health, future outcomes, and disease prevention, we want to emphasise their importance in the context of child development. The motor skills of speed, hand-eye coordination and flexibility are not only important for physical performance, but are also closely linked to various aspects of children’s daily lives. They influence participation in sports and recreational activities and overall physical wellbeing, making them an important area of study. Previous research has demonstrated the potential benefits of school-based PA interventions on PF (Barnett et al., 2009; Van Sluijs and McMinn, 2010; Lai et al., 2014; Brandes et al., 2023; Zhou et al., 2023), yet there remains a need for more comprehensive investigations that target specific age groups and assess multiple dimensions of motor performance. This study focuses on 8–to 9-year-olds and their development of speed, hand-eye coordination, and flexibility. Despite the relatively small impact of these components on health, our study aims to shed light on their multifaceted importance for children’s development and wellbeing. The purpose is to contribute to the existing body of knowledge on the effectiveness of school-based interventions to improve children’s PF.

The hypothesis of our study was that a 16-week school-based PA intervention would positively influence the PF of 8- to 9-year-olds. This study aims to shed light on the potential of such interventions to improve PA and fitness in young children and thus contribute to the formulation of evidence-based strategies to promote the general health and wellbeing of school-aged individuals.

## 2 Materials and methods

### 2.1 Subjects

The present study was a randomized experimental trial comparing an extracurricular PA program focusing on athletic and gymnastic activities with traditional PE. The study included 78 boys and girls (boys: n = 45, aged 8.4 ± 4.9 years; girls: n = 42, aged 8.6 ± 0.5 years) from the same school. Participants were excluded based on certain criteria, such as pre-existing medical conditions (heart disease, cancer, etc.). Overweight and obese individuals were included in this study. Randomization was performed using simple randomization. The participants were then divided into two groups: One group participated in an extracurricular PA program (EXP), while the other group formed the control group (CON) and participated only in mandatory PE classes. The general characteristics of the participants are shown in Table 1. Before the intervention began, all participants and their parents or guardians were familiarized with the experimental procedures and signed an informed consent form in order for the children to participate. The study procedures were conducted in accordance with the Declaration of Helsinki.

**TABLE 1:** General characteristics of the participants.

Variable	EXP group (*n* = 44)	CON group (*n* = 43)	*p*-value
BH (cm)	124.3 ± 6.6 (95% CI: 121.1–127.5)	125.5 ± 7.1 (95% CI: 122.2–128.8)	0.243
BW (kg)	24.3 ± 3.7 (95% CI: 23.1–25.6)	24.9 ± 4.1 (95% CI: 23.7–26.1)	0.312
BMI (kg/m^2^)	15.6 ± 0.9 (95% CI: 15.3–15.9)	15.7 ± 0.9 (95% CI: 15.4–16.0)	0.635
Age (years)	8.5 ± 0.5 (95% CI: 8.3–8.7)	8.6 ± 0.5 (95% CI: 8.4–8.8)	0.487

Abbreviations: BH, body height; BW, body weight; BMI, body mass index; EXP, experimental; CON, control; *n*, number of participants. Values are defined as mean ± standard deviation.

### 2.2 Procedures

Each test that was carried out took place in the morning and carried out by the same investigators. The tests included an examination of body composition and the developmental level of PF (speed, hand-eye coordination, and flexibility). Body height was measured to the nearest 0.5 cm using a wall-mounted stadiometer. Body weight was measured on a calibrated beam scale with an accuracy of 0.1 kg.

Both the EXP and CON groups participated in regular PE classes three times per week for 45 min each, with the EXP group engaging in additional extracurricular PA sessions twice per week. The additional sessions were based on athletic and gymnastic activities. The program was led by a PE teacher employed at the school where the intervention took place. The additional PA sessions were conducted for 16 weeks, with each session lasting 60 min. Each session began with approximately 5 min of warm-up training, which continued with running games from low to moderate to high intensity. This was followed by 10 min of stretching and strengthening exercises. The main part of the session lasted around 40 min and focused on athletic content (speed and hand-eye coordination) in one session per week and gymnastic content (hand-eye coordination and flexibility) in the other. Each session ended with 3–5 min of stretching and calming exercises. The program included several elementary PE games that included elements of athletics (skipping, push-off running, hopping, cross-stepping, and jumping, etc.) and gymnastics (handstand, forward roll, backward roll, hand support jumps, squat jump on a vault box, climbing on horizontal bars, incline benches and ropes, crawling, and jumping rope, etc.). These elements were identified as having the most positive impact on the development of speed, hand-eye coordination, and flexibility (Bade et al., 2023; Campbell-Pierre and Rhea, 2023; Moran et al., 2023). The additional PA sessions were conducted both indoors and outdoors, depending on the weather conditions. The CON group participated only in traditional PE classes or physical activities that were scheduled for all students during the academic day.

### 2.3 Physical fitness testing

Participants in this study underwent a series of PF tests, including the Sit and Reach Test (SAR), followed by the 30-meter sprint and subsequently the Alternate Hand Wall Toss Test (AHWT) at 1.0 and 2.0 m.

### 2.4 Sit and reach test (SAR)

Each participant was asked to sit on the flat floor of the gym and place their bare feet vertically against a box. In this starting position, they then had to lean forward as far as possible with their arms and knees fully extended and hold the final position for 5 s. Participants who did not fully extend their arms and knees in the final position or could not hold the position for 5 s repeated the measurement. Participants had 30 s rest between each trial, with their best result being recorded.

In evaluating the effectiveness and reliability of the SAR test, the study by Mayorga-Vega et al. (2014) plays a crucial role. They specifically assessed the concurrent validity of the SAR test, an important aspect in determining its accuracy in measuring flexibility. Their analysis resulted in a high intraclass correlation coefficient (ICC) of 0.93, which underlines the excellent concurrent validity of the test. This high ICC value not only confirms the reliability of the SAR test, but also its strong correlation with established benchmarks for assessing flexibility, validating its use in our study.

### 2.5 30-m sprint

Participants completed three 30-meter sprint trials from a standing start, with an interval of at least 5 min between each trial. Time was recorded to the millisecond for the first 30 m and measured with optical timing devices (Model: T-C System; Brower Timing Systems, Salt Lake City, UT).

The reliability and accuracy of the 30-metre sprint test is also underpinned by the study conducted by Simperingham et al. (2016), who specifically investigated the criterion-related validity of this test. Their comprehensive analysis revealed a high intraclass correlation coefficient (ICC) of 0.91. This significant result not only underlines the strong criterion-related validity of the 30-metre sprint test, but also demonstrates its consistency and robustness in line with the established criteria for measuring this particular physical attribute.

### 2.6 Alternate hand wall toss test 1.0 and 2.0 m (AHWT 1.0 and 2.0 m)

The AHWT is a test that measures hand-eye coordination. A tennis ball is thrown from one hand in an extended motion at a specified distance from the wall and an attempt is made to catch it with the other hand. The total number of repeated actions within 30 s is recorded. In this test, the distances were set at 1.0 and 2.0 m. First the ball was thrown with the right hand and caught with the left hand, then the ball was thrown with the left hand and caught with the right hand; this was recorded as 2 repetitions.

The precision and reliability of the AHWT test as a measure of hand-eye coordination is confirmed by the research of Cho et al. (2020). In their comprehensive study, they focused on evaluating the construct validity of the AHWT test. Their results, highlighted by an intraclass correlation coefficient (ICC) of 0.85, show a high degree of construct validity for the test. This robust ICC value is an indication of the effectiveness of the test in accurately measuring the specific construct of hand-eye coordination for which it was developed. This level of validation confirms the reliability of the test and its applicability in the given context and ensures the validity of the coordination measurements obtained in our study.

The reliability of the PF tests was determined in a preliminary study conducted by our research team that focused specifically on the same age group. In these preliminary results, all tests showed good reliability, with an intraclass correlation coefficient (ICC) ranging from 0.85 to 0.93.

### 2.7 Statistical analysis

Data analysis was performed with SPSS, version 23 (SPSS Inc., Chicago, IL, United States). Means and standard deviations were calculated for all variables. The normality of the data was confirmed with the Kolmogorov-Smirnov test (*p* > 0.05 for all tests), and the homogeneity of variances was assessed with the Levene test. A two-way repeated measures ANOVA was used to examine main effects and interactions for time (pre-test vs. post-test) and group (EXP vs. CON) on the selected outcomes. Following ANOVA, systematic *post hoc* paired-samples t-tests were conducted to assess within-group changes over time for each variable measured.

Cohen’s d was used to measure the effect size for within-group comparisons. The calculation of Cohen’s d was based on the standardized mean difference between the pre- and post-test scores in each group divided by the pooled standard deviation of both time points. This provides a measure of the size of the effect of the intervention in units of standard deviation. In our study, the effect size was categorised according to the guidelines of (Hopkins et al., 2009). In particular, a Cohen’s d value of less than 0.2 was interpreted as a trivial effect, 0.2–0.6 as a small effect, 0.6–1.2 as a moderate effect, 1.2–2.0 as a large effect, greater than 2.0 as a very large effect and greater than 4.0 as an extremely large effect. The partial Eta^2^ values (η^2^) were first calculated for the differences between the groups. These η^2^ values were then converted to Cohen’s d to ensure consistency in the quantification of effect sizes across the study. This conversion was particularly relevant in the context of significant interaction effects identified by the two-way repeated measures ANOVA. Once these interactions were identified, systematic *post hoc* paired-samples t-tests were conducted to assess within-group changes over time for each variable measured.

All statistical tests were two-tailed, and a *p*-value of ≤0.05 was considered statistically significant.

## 3 Results

**FIGURE 1:**
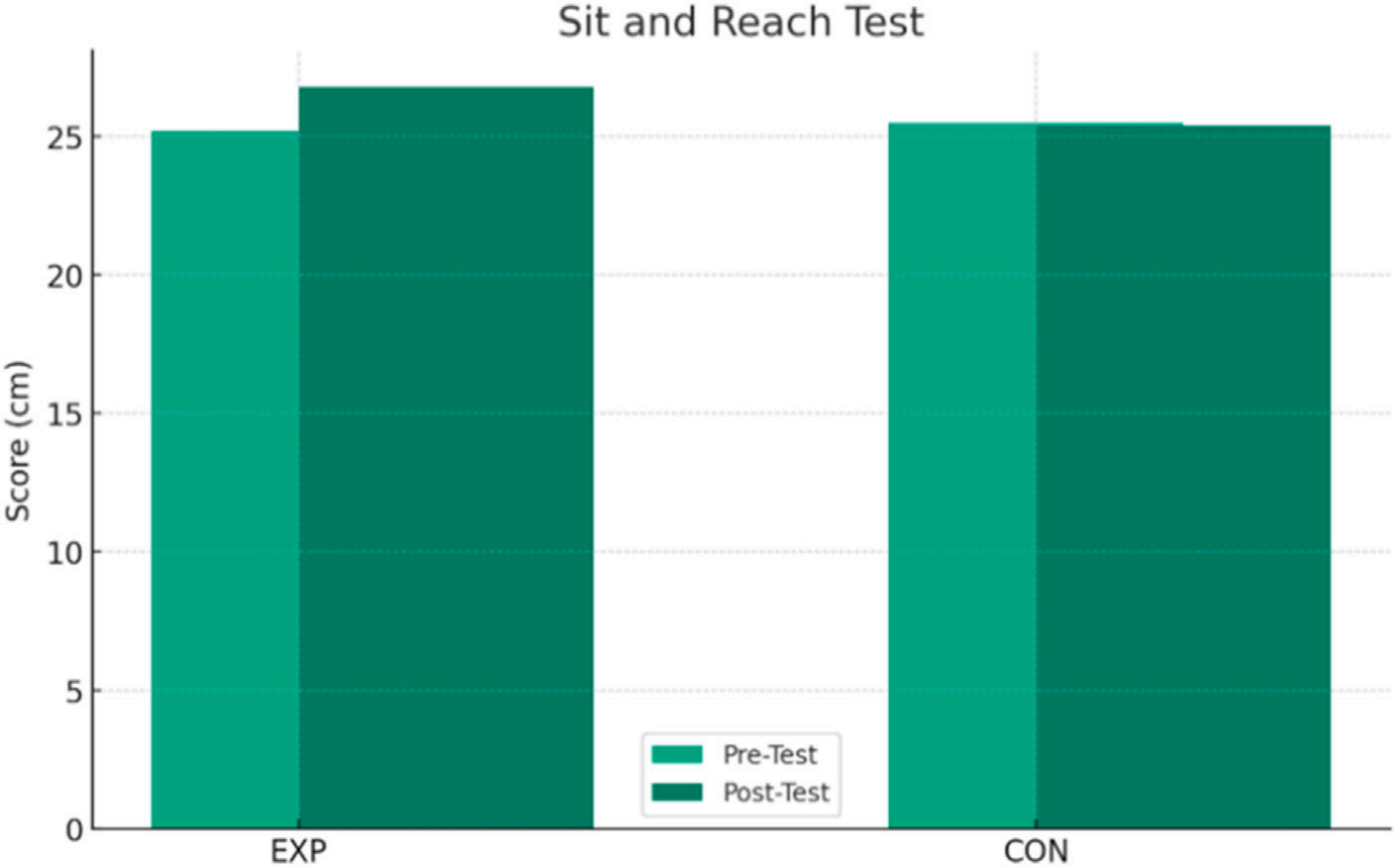
Change in the results of the Sit and Reach over time for the EXP and CON groups.

**FIGURE 2:**
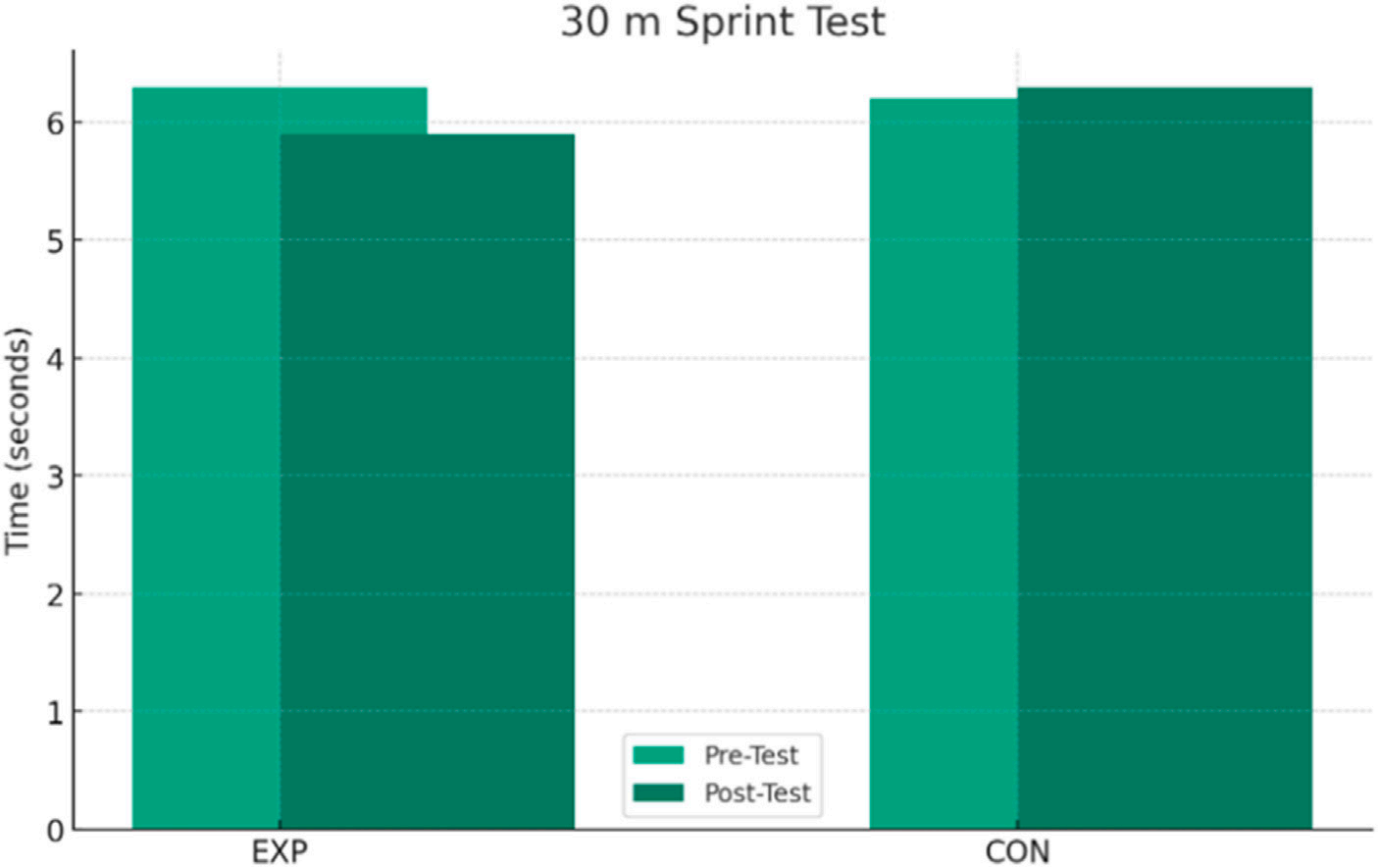
Change in 30 m sprint times over time for the EXP and CON groups.

**FIGURE 3:**
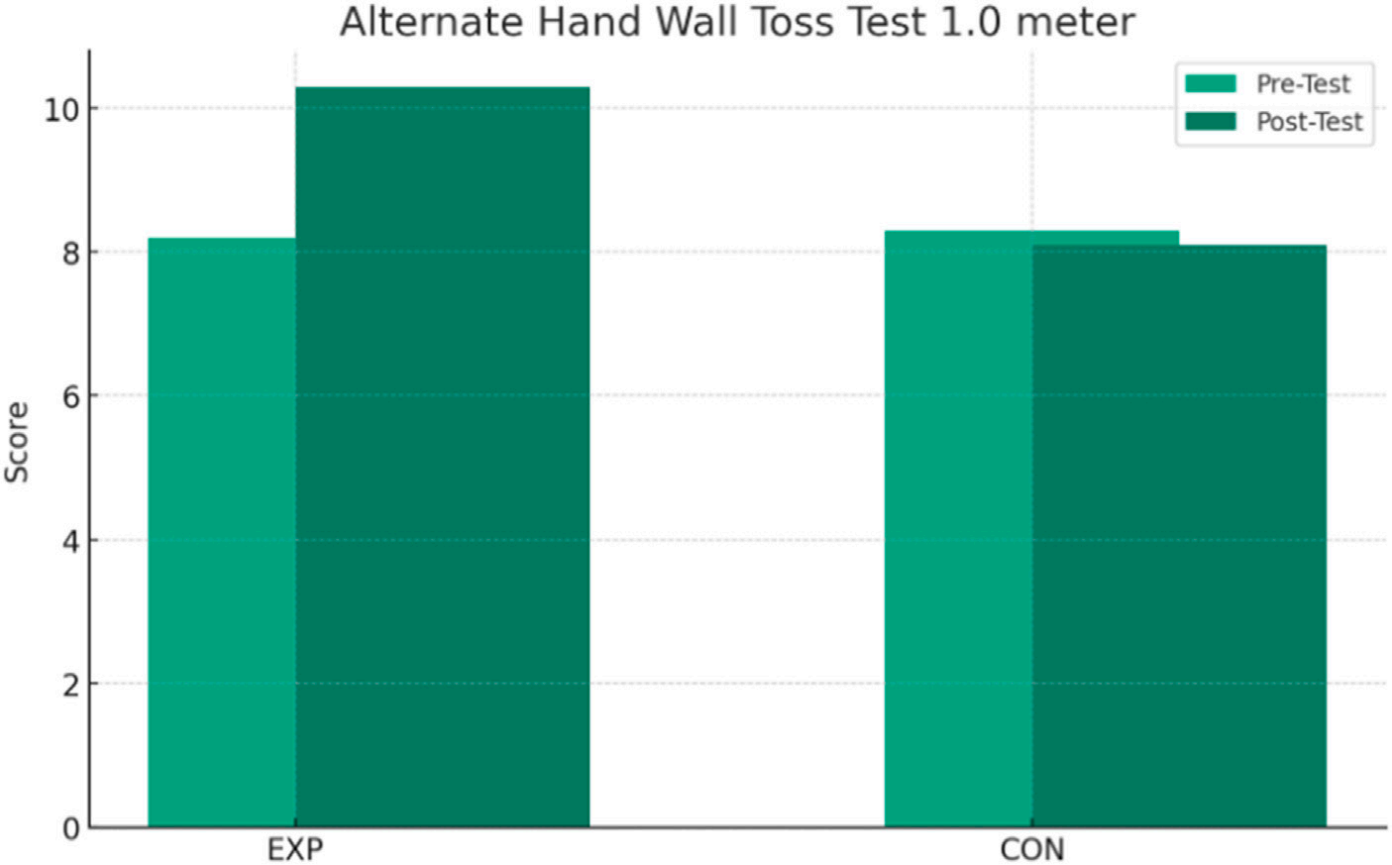
Change in results in the Alternate Hand Wall Toss Test 1.0 m over time for the EXP and CON groups.

The results presented in Table 2 show significant interactions between group and time for several tests. Post-hoc paired-samples t-tests within the EXP group showed a statistically significant improvement in the SAR test (*p* < 0.001), with an average increase of +1.6 cm from the pre-test (25.2 ± 3.3 cm) to the post-test (26.8 ± 3.7 cm) and an effect size (Cohen’s d) of +1.28, indicating a large effect (Figure 1). In addition, the EXP group showed a significant time reduction in the 30-metre sprint by −0.4 s on average (from 6.3 ± 0.5 to 5.9 ± 0.4 s), with an effect size of +0.67, representing a moderate effect, and a *post hoc p*-value of <0.001 (Figure 2). Significant improvements were also observed in the AHWT 1.0 test for the EXP group (Figure 3), with an average improvement of +2.1 points (from 8.2 ± 3.7 to 10.3 ± 3.9) and an effect size of +1.78, reflecting a very large effect (*post hoc p*-value <0.001).

**TABLE 2:** Physical fitness results and changes from pre- to post-test in EXP and CON group.

Variable	Group	Pre-test	Post-test	ES	% Change	*p*-value, η^2^_p_	Post-hoc test (paired *t*-test *p*-value)
Sit and reach test (cm)	EXP	25.2 ± 3.3	26.8 ± 3.7	+1.28	+6.3	Group: *p* = 0.469, η^2^_p_: 0.006 Time: *p* < 0.001, η^2^_p_: 0.306 Interaction: *p* < 0.001, η^2^_p_: 0.361	*p* < 0.001
	CON	25.5 ± 3.0	25.4 ± 3.5	−0.09	−0.4		*p* = 0.533
30 m sprint (seconds)	EXP	6.3 ± 0.5	5.9 ± 0.4	+0.67	+6.3	Group: *p* < 0.177, η^2^_p_: 0.021 Time: *p* < 0.001, η^2^_p_: 0.179 Interaction: *p* < 0.001, η^2^_p_: 0.193	*p* < 0.001
	CON	6.2 ± 0.6	6.3 ± 0.6	−0.22	−1.6		*p* = 0.150
Alternate hand wall toss test 1.0 m (score)	EXP	8.2 ± 3.7	10.3 ± 3.9	+1.78	+25.6	Group: *p* < 0.241, η^2^_p_: 0.016 Time: *p* < 0.001, η^2^_p_: 0.456 Interaction: *p* < 0.001, η^2^_p_: 0.545	*p* < 0.001
	CON	8.3 ± 4.0	8.1 ± 4.6	−0.21	−2.4		*p* = 0.186
Alternate hand wall toss test 2.0 m (score)	EXP	3.8 ± 3.6	3.7 ± 3.9	−0.13	−2.6	Group: *p* = 0.949, η^2^_p_: 0.001 Time: *p* = 0.254, η^2^_p_: 0.015 Interaction: *p* = 0.888, η^2^_p_: 0.001	*p* = 0.400
	CON	3.8 ± 3.6	3.7 ± 4.2	−0.12	−2.6		*p* = 0.430

Abbreviations: EXP, experimental group; CON, control group; ES, cohen d effect size; Values are defined as mean ± standard deviation.

**FIGURE 4:**
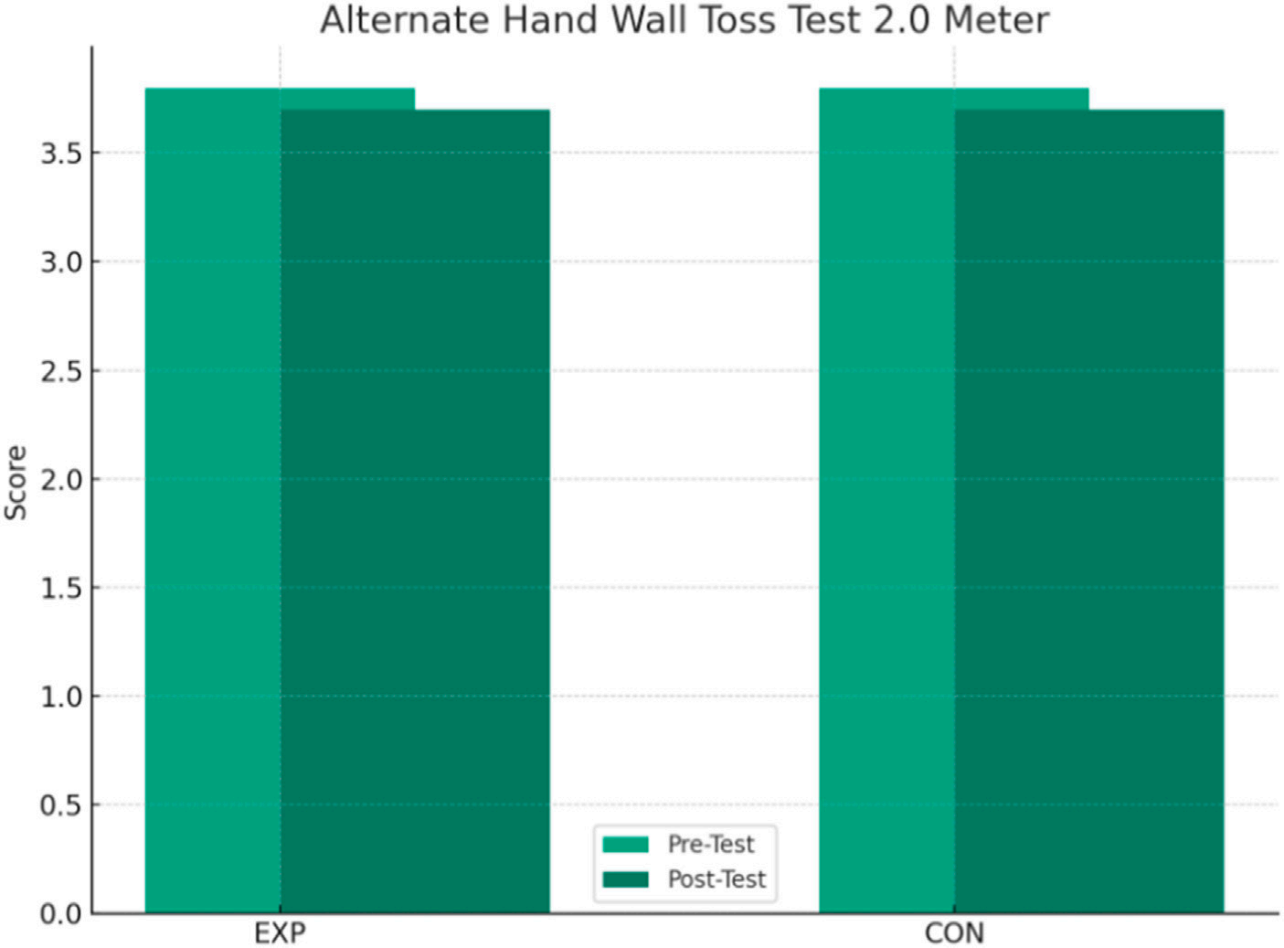
Change in results in the Alternate Hand Wall Toss Test 2.0 m over time for the EXP and CON groups.

In contrast, the *post hoc* analyses within the CON group showed no statistically significant changes over time for the measures with interaction. In particular, the *p*-values for SAR test (*p* = 0.533), 30-m sprint (*p* = 0.150), AHWT 1.0 test (*p* = 0.186), and AHWT 2.0 test (*p* = 0.430) were non-significant differences, indicating a clear change over time in the CON group. The interaction between group and time for AHWT 2.0 test was not significant (*p* = 0.888), with both groups showing a slight decrease in scores, suggesting that the intervention did not lead to significant improvements on this measure (Figure 4).

There were no dropouts in either the EXP or CON groups throughout the study period, demonstrating the feasibility and tolerability of the intervention program.

## 4 Discussion

Incorporating interventions that aim to improve the PF of speed, hand-eye coordination, and flexibility is particularly important during the developmental period of 8–to 9-year-olds. This is because improvements made during this period can have a positive impact on a child’s lifelong physical health and wellbeing (Stanković et al., 2022). Acquiring PF not only contributes to performance in sports and physical activities, but also promotes a holistic understanding of their bodies and the value of an active lifestyle (Baena-Morales and González-Víllora, 2023). Therefore, the aim of this study was to examine the impact of a 16-week extracurricular intervention program focusing on PA and the impact it had on the PF of 8–to 9-year-olds. A key finding of this study was that the EXP group showed remarkable improvements in speed, hand-eye coordination, and flexibility following the concise program. In addition, the 16-week school-based PA intervention effectively improved PF in all studied domains except AHWT 2.0 m test, where no differences between groups were recorded.

Lack of flexibility in children aged 8–9 years can hinder their physical development by limiting their range of motion and predisposing them to musculoskeletal problems (Bauer et al., 2021; Zmyślna et al., 2021). It is important to address this issue as promoting proper flexibility at this age improves the overall efficiency of movement. Previous studies (Radulović et al., 2022; Xu et al., 2022; Kryst et al., 2023) have shown that flexibility decreases significantly between the ages of 7 and 15 (1996–2011). Subsequently, boys’ scores steadily declined and reached their lowest point in the year of 2019, while the studies show that girls reached their highest observed flexibility throughout the years of 1989–2019 (Radulović et al., 2022). Studies that included flexibility-targeted interventions, such as stretching programs or specific PE curricula, have shown improvements in SAR test scores over specific time periods (Kumar and Zemková, 2022; Stamenković et al., 2022; Wang et al., 2022). The results of this study suggest that flexibility can be effectively improved through a school-based PA intervention, resulting in a notable 6.3% increase in performance SAR test. The flexibility of the children in this study increased statistically and significantly in the EXP group; however, it should be noted that the observed increase after the 16-week intervention in the supplementary program was somewhat lower than originally expected. This result can be attributed to several factors. One important factor is the role of muscle extensibility and suppleness. Both extensibility and suppleness are essential for optimal joint function and mobility. During the intervention, these muscle properties were specifically trained and improved, which contributed to improved flexibility. Advances in flexibility have been shown to reduce the risk of musculoskeletal injury by ensuring that joints can move through their full intended range without strain as proven in the study by Zvetkova et al. (2023). In addition to the two additional hours of PA in school for the EXP group, the difference in flexibility improvement between the EXP and CON group, in which the score decreased by 0.4% instead of improving, could be attributed to the nature of the program content. The program offered to the EXP group may have been more engaging and effective compared to traditional PE. As mentioned, each session was based around athletic and gymnastic exercises; however, these were conveyed through games, which could have influenced the children’s motivation and consequently resulted in a higher number of repetitions.

The results of the 30-m sprint test were also similar, as the students in the EXP group improved by 6.3%, just as they did in the SAR test, while there was a slightly larger decrease in the CON group, where the students dropped in speed by 1.6%. The result was not a surprise, as a greater progress in the students who participated in the supplemental program was expected than in those who participated only in regular PE. This was predicted because improvements in speed as a PF are often influenced by the specificity of the training program, and in the EXP group, the teacher tried to adjust the activities and games in each lesson to focus on specific components of speed, such as acceleration, agility, or sprint mechanics, which led to an improvement in overall speed performance. Similar findings were obtained by Mijalković et al. (2022), who investigated school-based exercise programs to promote cardiorespiratory fitness in overweight and obese children aged 6–10 years. In their study, the implementation of structured exercise programs potentially contributed to the improvement of the participants’ speed. This underscores the importance of exercise programs that are tailored to specific age groups and fitness components. Additionally, Wang et al. (2022), conducted a study that examined the effects of an 8-week neuromuscular training (NMT) method combined with regular tennis training in beginner tennis players aged 7–8 years old. In the 30-m sprint test, they recorded statistically significant differences (*p* = 0.001) in speed within the EXP group. This suggests that the combination of structured PA and potentially sensitive periods in child development may contribute to improved speed performance in this age group. However, on the other hand, Stojanović et al. (2023) compared the effects of a 16-week Teaching Games for Understanding (TGfU) volleyball intervention in students aged 13 ± 3.7 years, and also found a statistically significant improvement in speed in the 30-m sprint test (*p* = 0.019).

In this study, slightly different results were obtained when examining the effects of the intervention on hand-eye coordination development in children. The AHWT test, which was divided into two parts, AHWT 1.0 m and AHWT 2.0 m test, was used to investigate this depending on the level of difficulty. In the easier version, students stood 1 meter away from the wall during the test and statistically significant differences in hand-eye coordination development was found between the EXP and CON groups. In particular, the result of the EXP group improved by 25.6%. This result is quite high, suggesting that the participants may not have exerted much effort on the initial test, despite precise demonstration and explanation by the teacher. However, the significant gains in hand-eye coordination observed in the EXP group may indicate that the intervention program not only improved their hand-eye coordination, but also enhanced the development of their ability to handle a tennis ball with greater dexterity and precision. This multifaceted improvement underscores the potential of well-structured programs that not only target specific fitness components, but also promote related motor skills and coordination. The comprehensive impact of such interventions on the overall development of participants is a notable finding of this study. Compared to the EXP group, the CON group had poorer results, deteriorating by an average of 2.4% from the pre-test to the post-test. The more challenging version of the hand-eye coordination development test, in which all participants performed the same movements as in the first part of the test but were positioned 2 m away from the wall, resulted in significantly lower pre-test scores. Neither the EXP nor the CON group showed any improvement on the test; instead, both groups experienced a 2.6% decrease in performance. In this context, Frikha and Saad Alharbi (2023) conducted an intervention aimed at improving fine motor coordination, selective attention, and reaction time in children aged 8.29 ± 0.74 years through precision motor exercises. In the EXP group, hand-eye coordination improved significantly (*p* < 0.05). In contrast to this study, where there was no statistically significant improvement in the AHWT 2.0 test even within the EXP group after the intervention, Kahana et al. (2022) observed statistically significantly higher results (*p* < 0.0001) in children aged 10 by an intervention in which a training application was introduced (APP). Despite the potential of this study’s PA intervention to improve various aspects of PFin children, the complicated nature of hand-eye coordination development might require more targeted and prolonged interventions to show remarkable improvements. In addition, individual differences in baseline hand-eye coordination skill and the complexity of the coordination process itself could contribute to the difficulty of achieving significant improvements through general PA interventions.

Although a significant improvement in PF was observed, the study acknowledges certain limitations. While the selection of participants aged 8–to 9-years ensured homogeneity within the study group, it did not consider differences in biological age that may affect physical development and performance. This is a potential limitation as biological age may differ significantly from chronological age, especially during a developmental phase. Future research could benefit from the inclusion of measures of biological maturity to better understand the effects of PA interventions. In addition, the study did not measure participants’ enjoyment and satisfaction, which could influence the effectiveness of the intervention. Assessing participant engagement and enjoyment could provide valuable insights for future PA programs. Furthermore, the lack of objective accelerometer data to measure students’ overall PA during the intervention period could limit the interpretation of the results. Despite these considerations, this study highlights the value of school-based PA programs, which appear to significantly improve PF in 8- to 9-year-olds compared to traditional PE, particularly in an age group where there has been a marked decline in moderate to vigorous PA.

## 5 Conclusion

The results of this study indicate that a 16-week school-based PA program can significantly improve speed and flexibility, or musculoskeletal fitness compared to traditional PE. In addition, hand-eye coordination was also improved as measured by the 1.0-meter AHWT test. An important finding of our research is that remarkable progress can be made in certain aspects of PF within a relatively short period of time. However, this was not true for all measures of fitness, as the 2.0-meter AHWT test showed no significant improvement. These results suggest that the development of PF in younger students can be significantly improved by introducing just two additional hours of weekly PA for some fitness components, but that this is not equally true for all aspects of PF. While the results support the integration of additional PA into the curriculum, they also highlight the need for a differentiated approach tailored to the different components of PF.

## Data Availability

The raw data supporting the conclusion of this article will be made available by the authors, without undue reservation.
